# Leo XIII.—Pius X

**Published:** 1903-09

**Authors:** 


### Leo XIII.—Pius X.

THE death of Pope Leo XIII., which occurred July 20, 1903, terminated the reign of one of the wisest pontiffs that has ever ruled the catholic church at Rome. As a member of the house of Pecci, when elected to the papal throne, he may be said to have represented the nobility when chosen to the high office which he administered so ably for 25 years. During his incumbence the catholic church made steady progress toward an improved order of administration. The illness of the great pontiff was as pathetic as it was remarkable. He maintained a cheerful spirit to the last. His malady was unusual for one of his years, and he bore the repeated operative procedures for the removal of accumulation of pleuritic fluid with complacent resignation. Dr. Lapponi, the faithful physician, who conducted the case, acquitted himself throughout the trying ordeal with dignity and skill.

The conclave appears to have chosen wisely in the election of Cardinal Sarto, the patriarch of Venice, who announces himself as Pius X. Though of peasant origin he is a learned man, with a taste for music and art, and bids fair to carry forward the work of the church intrusted to his care, on the lines pursued by his predecessor.

